# Unveiling the K^+^-sensitivity of cell metabolism using genetically encoded, FRET-based K^+^, glucose, and ATP biosensors

**DOI:** 10.1016/j.xpro.2021.100843

**Published:** 2021-09-20

**Authors:** Helmut Bischof, Sandra Burgstaller, Wolfgang F. Graier, Robert Lukowski, Roland Malli

**Affiliations:** 1 Department of Pharmacology, Toxicology and Clinical Pharmacy, Institute of Pharmacy, University of Tuebingen, Auf der Morgenstelle 8, 72076 Tuebingen, Germany; 2 Gottfried Schatz Research Center, Molecular Biology and Biochemistry, Medical University of Graz, Neue Stiftingtalstraße 6/6, 8010 Graz, Austria; 3 Natural and Medical Sciences Institute, University of Tuebingen, 72770 Reutlingen, Germany; 4 BioTechMed-Graz, Mozartgasse 12/II, 8010 Graz, Austria

**Keywords:** Cancer, Cell Biology, Cell culture, Chemistry, Metabolism, Microscopy, Molecular Biology, Molecular/Chemical Probes

## Abstract

Investigating dynamic changes of mitochondrial ATP and cytosolic glucose levels of single living cells over time by genetically encoded biosensors provides an informative readout of their metabolic activities. Here, we describe how to monitor the metabolic K^+^-sensitivity of HEK293 cells exploiting ATP-, glucose-, and K^+^ probes. Fluorescence live-cell imaging of these Förster resonance energy transfer-based biosensors over time in response to gramicidin, an ionophoric peptide, indicated an absolute dependency of cellular ATP homeostasis on high intracellular K^+^ levels.

For complete information on the generation and use of this protocol please refer to Bischof et al. (2021).

## Highlights

• Single live-cell imaging allows the metabolic phenotyping of (cancer) cell lines
• FRET-based probes allow high-temporal resolution imaging
• Protocol allows us to investigate the K^+^ sensitivity of (cancer) cell metabolism

## Graphical abstract

**Figure:**
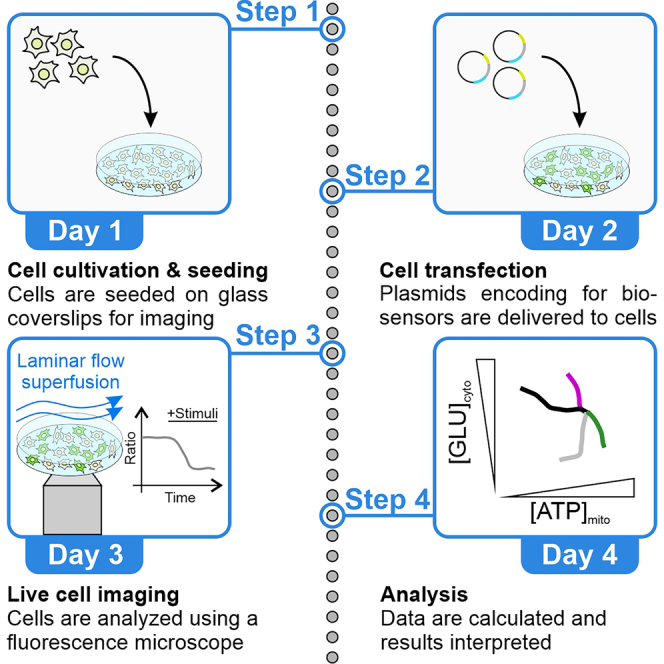

## Before you begin

The workflow described in this protocol takes several days and requires preparation and access to special equipment (see materials and equipment). Before starting the experiment, buffers and media should be prepared.

***Note:*** As the optimal conditions for cell growth, we suggest to follow the guidelines recommended by the provider of the cell lines. Accordingly, the cell number, cell culture medium, supplements, and times for preparing cells that express the genetically encoded probes indicated in this protocol might also vary depending on the cell type used. This protocol will describe the specific steps for HEK293 cells. However, we have also used this protocol with other cell lines including HeLa, INS-1 832/13, MCF-7 or MDA-MB-453 cells. For each cell line, a 10 cm cell culture dish at a confluency of ∼90% yielded enough cells for several experiments. For other cell lines, we expect, as mentioned above, that only minor adjustments of some experimental procedures will be necessary.

### Purification of sensor plasmids from *E.coli*

**Timing: Day 0**

1. Sufficient amounts of the plasmids FLII12Pglu-700μδ6 (Takanaga et al., 2008), mtAT1.03 (Imamura et al., 2009), mt lc-LysM GEPII 1.0 and NES lc-LysM GEPII 1.0 (Bischof et al., 2017) encoding for the respective genetically-encoded, FRET-based probes need to be prepared before the experiments.

***Note:*** Conventional plamid Maxiprep systems including an endotoxin removal step can be used for DNA purification. DNA preparations should be performed according to the manufacturer’s instructions. Upon plasmid purification, the concentration of the DNA (μg/μL) should be determined and can be stored at 4°C for up to 6 months or at −18°C for up to 1 year.

**CRITICAL:** The DNA purity needs to be checked (Lucena-Aguilar et al., 2016), as RNA or protein contaminations might affect transfection efficiency or cell viability. After the DNA purification, the DNA should importantly be verified by sequencing to check for potential DNA mutations affectingsensor functionalities.

### Culture of cell lines

**Timing: Day 0**

2. HEK293 cells utilized in this protocol were cultivated in DMEM + 10% fetal calf serum (FCS) + 1**×** penicillin-streptomycin + 1**×** sodium pyruvate. Cells were cultivated in a humidified incubator at 37°C and 5% CO_2_. Cell splitting and trypsinization were performed when cells reached a confluency of 90% using 0.05% Trypsin-EDTA in PBS.

**CRITICAL:** Any disturbances that could potentially influence cell metabolism and –activities (temperature fluctuations, CO_2_ fluctuations, altering the nutrient composition of the medium,…) should be avoided as far as possible.

### Preparation of the assay buffers

**Timing: Day 2**

3. For the metabolic phenotyping of the different cell lines by fluorescence microscopy using genetically-encoded probes, different buffers are required. The composition of these buffers can be found in the “materials and equipment” section below.

## Key resources table

**Table:** 

REAGENT or RESOURCE	SOURCE	IDENTIFIER
**Chemicals, peptides, and recombinant proteins**		

3-Bromo-2-oxopropionic acid (3-BP)	Sigma Aldrich	N/A
50**×** MEM Amino Acids Solution	Thermo Fisher Scientific	N/A
100**×** MEM Vitamin Solution	Thermo Fisher Scientific	N/A
100**×** Sodium Pyruvate Solution	Thermo Fisher Scientific	N/A
Antimycin-A	Abcam	N/A
CaCl_2_ ⋅ 2H_2_O	Carl Roth	N/A
D-Glucose ⋅ H_2_O	Carl Roth	N/A
DMEM, high Glucose, HEPES	Thermo Fisher Scientific	N/A
Fetal Calf Serum	Thermo Fisher Scientific	N/A
Gramicidin	Sigma Aldrich	N/A
HEPES	Carl Roth	N/A
KCl	Carl Roth	N/A
KH_2_PO_4_	Carl Roth	N/A
L-Glutamine (200 mM)	Thermo Fisher Scientific	N/A
MgCl_2_ ⋅ 6H_2_O	Carl Roth	N/A
NaCl	Carl Roth	N/A
NaHCO_3_	Carl Roth	N/A
NaH_2_PO_4_	Carl Roth	N/A
Na_2_HPO_4_	Carl Roth	N/A
NaOH	Carl Roth	N/A
Oligomycin-A	Tocris	N/A
Penicillin-Streptomycin (100**×**)	Thermo Fisher Scientific	N/A
Phosphate Buffered Saline	Thermo Fisher Scientific	N/A
Trypsin-EDTA (0.5%)	Thermo Fisher Scientific	N/A

**Experimental models: cell lines**		

Human: HEK293	ATCC	N/A

**Recombinant DNA**		

mt lc-LysM GEPII 1.0, K^+^ sensor targeted to the mitochondrial matrix	Next Generation Fluorescence Imaging GmbH	N/A
NES lc-LysM GEPII 1.0, K^+^ sensor targeted to the cytosol	Next Generation Fluorescence Imaging GmbH	N/A
pcDNA3.1 FLII^12^Pglu-700μδ6, glucose sensor targeted to the cytosol	Addgene	N/A
mtAT1.03, ATP-sensor targeted to the mitochondrial matrix	Imamura et al.	N/A

**Software and algorithms**		

Excel Office 365	Microsoft	N/A
Prism 5	GraphPad	N/A
VisiView	Visitron Systems	N/A

**Other**		

6-well plates	Corning	N/A
15 mL Centrifuge tubes	Corning	N/A
30 mm circular glass coverslips	Paul Marienfeld GmbH & Co. KG, Lauda-Königshofen, Germany	N/A
37°C incubator, 5% CO_2_, humidified	N/A	N/A
37°C water-bath	N/A	N/A
Bürker-Türk cell counting chamber	N/A	N/A
Centrifuge	N/A	N/A
Omicron LEDHub	Omicron, Klaus, Austria	N/A
xcitation filter	IDEX Health & Science, Rochester, NY, USA	N/A
Emission filter	IDEX Health & Science, Rochester, NY, USA	N/A
Multichannel perfusion system	NGFI GmbH, Graz, Austria	N/A
Optosplit II Image Splitter	Cair Research, Faversham, UK	N/A
PC	N/A	N/A
Pco.panda 4.2 bi	PCO AG, Kelheim, Germany	N/A
Perfusion Chamber PC30	NGFI GmbH, Graz, Austria	N/A
Perfusion System PS-9D	NGFI GmbH, Graz, Austria	N/A
Sterile work bench	N/A	N/A
Vacuum pump	N/A	N/A
Zeiss Axio Observer Z1	Carl Zeiss AG, Oberkochen, Germany	N/A

## Materials and equipment

We highly recommend preparing stock solutions in ddH_2_O to minimize inaccuracies.

**Table:** Formulation of cell equilibration buffer

Reagent	Final concentration	Amount
CaCl_2_ ⋅ 2H_2_O	2 mM	20 mL of 0.1 M
D-Glucose ⋅ H_2_O	10 mM	10 mL of 1 M
HEPES	10 mM	1 mL of 1 M
KCl	5 mM	50 mL of 0.1 M
l-Glutamine	2 mM	10 mL of 100**×**
KH_2_PO_4_	0.44 mM	4.4 mL of 0.1 M
MgCl_2_ ⋅ 6H_2_O	1 mM	10 mL of 0.1 M
NaCl	135 mM	135 mL of 1 M
NaHCO_3_	2.6 mM	2.6 mL of 1 M
Na_2_HPO_4_	0.34 mM	3.4 mL of 0.1 M
MEM amino acids	1**×**	20 mL of 50**×**
MEM vitamins	1**×**	10 mL of 100**×**
Penicillin-Streptomycin	1**×**	10 mL of 100**×**
ddH_2_O	-	to 1 L
**Total**	**N/A**	**1 L**

After mixing all components, the pH should be adjusted to 7.4 using NaOH. The cell equilibration buffer should be sterile filtered and can be stored at 4°C in the dark for up to 2 months.

**Table:** Formulation of glucose free imaging buffer (IB – GLU)

Reagent	Final concentration	Amount
CaCl_2_ ⋅ 2H_2_O	2 mM	20 mL of 0.1 M
HEPES	10 mM	1 mL of 1 M
MgCl_2_ ⋅ 6H_2_O	1 mM	10 mL of 0.1 M
NaCl	138 mM	135 mL of 1 M
ddH_2_O	-	to 1 L
**Total**	**N/A**	**1 L**

After mixing all components, pH should be adjusted to 7.4 using NaOH. Nutrient-free imaging buffer can be stored at 4°C for up to 1 week.

## Step-by-step method details

### Cell counting and seeding

**Timing: Day 1, 1 h**

Handling the cells for this experiment does not require special treatment. We recommend optimizing the seeded cell number empirically.

***Note:*** The following steps will describe the protocol for using HEK293 cells. The protocol might be adjusted for the use with other cell lines. All media and buffers getting in contact with the cells should be prewarmed to 37°C using a water bath.

1. Remove the cells cultivated within a 10 cm dish to a confluency of ∼90% from the incubator and detach the cells under sterile conditions.
  a. Dilute Trypsin-EDTA to the desired final concentration with PBS. Typically, 0.05% of Trypsin-EDTA is sufficient for the trypsinization of HEK293 cells.
  b. Put the plate under the sterile cell culture hood, remove the cell culture medium, and replace the cell culture medium twice with 10 mL of pre-warmed PBS.
  c. Remove PBS and add 3 mL of a prewarmed, 0.05% Trypsin-EDTA in PBS.
  d. Put the cells back into the incubator and check for cell detachment after 2 min. If cells have not detached after 5 min, keep incubating them at 37°C and check for detachment every 1 min.
  e. When cells are floating, stop trypsinization by adding 10 mL of pre-warmed complemented medium (DMEM + 10% FCS + 1**×** penicillin-streptomycin + 1**×** sodium pyruvate).
  f. Transfer the cells to a 15 mL conical tube and pellet them at 200 × g for 5 min.
  g. After centrifugation, remove the supernatant from the cells without disturbing the cell pellet and fully resuspend the cell pellet in 10 mL of complemented-medium (DMEM + 10% FCS + 1**×** penicillin-streptomycin + 1**×** sodium pyruvate) carefully.
2. Determine the cell number/milliliter by counting the cells using a Bürker-Türk counting chamber or any other method of choice.
  a. When using a Bürker-Türk counting chamber, use 15 μL of cell suspension for one chamber. We recommend the determination of the cell number in duplicates by counting 2 × 4 large squares.
  b. For each of the two chambers, divide the number of total cells counted in the 4 large squares by 4 and take the average of the two chambers.
  c. Multiply the number of cells × 10.000, which gives you the cell number/milliliter.
3. Seed HEK293cells into the desired number of wells in a 6-well plate containing 30 mm circular glass coverslips and cultivate the cells for 16–24 h.
  a. Prepare the desired number of 6-well plates.
    i. Put 30 mm circular glass coverslips in the desired number of wells in 6-well plates.
    ii. Add 1 mL of complemented medium (DMEM + 10% FCS, + penicillin/ streptomycin, + 1**×** sodium pyruvate) and distribute the medium in the wells.
    iii. Using a sterile pipette tip, push the coverslips to the bottom of the 6-well plate to ensure that the coverslips firmly stick to the bottom of the plate to prevent cell growth beneath the glass coverslip.
  b. Dilute the cells to an appropriate cell number/milliliter and seed into the wells of the 6-well plate containing the glass coverslips.
    i. The cell number that needs to be seeded varies with the time between seeding the cells and performing the experiment. For HEK293 cells that should be investigated 2 days after seeding, typically 300.000 cells per well are sufficient to achieve a density of approximately 70–80% on the day of the experiment, which is ideal for the assays.
    ii. Ideally, the final culture volume per well is 1.5 mL. Dilute the cell number of the HEK293 cells to 600.000/mL and add 500 μL of the cell suspension to each well of the 6-well plate containing the glass coverslip, yielding a final culture volume of 1.5 mL and a cell number of 300.000 cells/well.
  c. Put the 6-well plates into a humidified incubator with 37°C and 5% CO_2_ for 16–24 h and cultivate the cells.

### Cell transfection with the sensor plasmids

**Timing: Day 2, 1 h**

The genetic information of the sensors, i.e., the plasmids, needs to be delivered to the cells. Therefore, different transfection methods, electroporation, or viral transduction may be used.

***Note:*** In this protocol, we describe how to transfect HEK293 cells using the PolyJet transfection reagent. Depending on the transfection reagent, the protocol may vary, and other reagents/methods for plasmid delivery might be required for different cell types.

4. Perform a medium change of the cells seeded the day before.
  a. Remove the cells from the incubator (humidified, 37°C, 5% CO_2_) and check the cells using a cell culture microscope for their morphology, density, and possible bacterial or fungal contaminations.
    i. If the cells should be measured at the fluorescence microscope the next day, HEK293 cells should show a density of approximately 40%–50% on the day of the transfection.
  b. Remove the medium from each well that will be transfected with a sensor plasmid and carefully add 1 mL of fresh and prewarmed complemented medium (DMEM + 10% FCS, + penicillin/ streptomycin, + 1**×** sodium pyruvate) to each well.
  c. After changing the medium, return the cells to the incubator for 30 min.
5. Prepare the transfection mixture using the plasmid of choice and PolyJet transfection reagent. For PolyJet transfection also see: https://signagen.com/DataSheet/SL100688.pdf
  a. Approximately 20 min after putting the cells back into the incubator, start with preparing the transfection mixture.
    i. Therefore, 50 μL of FCS-free DMEM medium are prepared for each well that will be transfected with a plasmid. Hence, if two 6-well plates will be transfected with two different probes (probe A and B), 2 × 300 μL of serum-free DMEM are required and put in separate tubes (tubes A and B). Per transfected well, 1.0 μg of plasmid DNA should be added. For mitochondria-targeted constructs, the DNA amount should be reduced to 0.3 μg/well to ensure proper mitochondrial targeting of the probes.
    ii. For each well that will be transfected, 50 μL of FCS-free DMEM are prepared in a separate tube (tube C) for the transfection reagent. Hence, if two 6-well plates will be transfected (this is independent of the number of different plasmids), 600 μL of serum-free DMEM are pipetted in tube C. Subsequently, 3 μL of PolyJet transfection reagent are added for each well that should be transfected and the tube is mixed by pipetting.
    iii. After the DNA (tube A and B) and PolyJet transfection reagent (tube C) were prepared, the DNA-containing medium and PolyJet-medium mixtures are mixed at a 1:1 ratio. Hence, if 300 μL of the DNA-medium mixture are in tube A, 300 μL of the PolyJet-media mixture from tube C are added, followed by thorough mixing by pipetting. The same applies to the second sensor in tube B, which is also mixed in a 1:1 ratio with 300 μL of tube C. Mixtures are incubated for 10 min at 20°C–25°C.
  b. After 10 min of incubating the DNA-transfection mixtures, the transfection mixtures are added to the cells.
    i. The cells are removed from the incubator and 100 μL of each transfection mixture is added in a drop-by-drop manner to each well of the 6-well plate that should be transfected with the respective probe.
    ii. Once the mixtures are added, the cells are returned to the incubator and incubated at 37°C with 5% CO_2_ for 12–16 h.

***Note:*** Depending on the cell type used, other transfection reagents, incubation times, plasmid- or transfection reagent amounts may be required.

**CRITICAL:** The transfection mixture containing the transfection reagent and the plasmid DNA must be prepared in FCS free medium. Furthermore, after mixing the reagent and plasmid DNA together, the vial should incubate without perturbations to allow the transfection complex to form.

### Preparing cells for the experiments

**Timing: Day 3, 20 min**

On the day of an experiment, the cells should be removed from the incubator and the transfection mixture is replaced with the cell equilibration buffer. This allows the cells to acclimate to the environment of the recording conditions, which ensures data consistency.

6. 12–16 h after adding the transfection mixture to the cells, the transfection mixture should be removed and cells should be equilibrated in the cell equilibration buffer.
  a. Pre-warm PBS and the cell-equilibration buffer to 37°C using a water bath.
  b. Remove the cells from the incubator and check the morphology, density, and possible contaminations using a cell culture microscope.
  c. Exchange the medium of the cells for the cell-equilibration buffer.
    i. Remove all medium from the cells.
    ii. Carefully replace the medium 1**×** with PBS by adding 1.5 mL of PBS / well to remove any residual medium.
    iii. Subsequently, remove the PBS and carefully add 1.5 mL/well of the cell-equilibration buffer. Keep cells in the dark at 20°C–25°C until they are measured. We recommend pre-equilibrating the cells for at least 30 min to allow their adaption to their new environment.

***Note:*** Cell pre-equilibration with the cell-equilibration buffer may be omitted if desired and cells might directly be measured from the incubator.

***Optional:*** The cells can also be cultivated for a longer period after their transfection (e.g. additional 24 h). In such cases, the transfection mixture should be exchanged for fresh complemented medium (DMEM + 10% FCS + 1**×** penicillin-streptomycin + 1**×** sodium pyruvate) after 12–16 h. The cells can then be further cultivated in DMEM + 10% FCS + 1**×** penicillin-streptomycin + 1**×** sodium pyruvate until the experiments will be conducted.

**CRITICAL:** It is important to remove the cationic lipids contained in the transfection mixture after 16 hours as a prolonged exposure will increase the toxcicity of the mixture.

### Complementing buffers for the experiments

**Timing: Day 3, 30 min**

Depending on the information that should be obtained from the experiments, a nutrient-free imaging buffer should be complemented with specific nutrients or modulators of cell metabolism such as inhibitors/activators of oxidative or glycolytic activity.

***Note:*** In this protocol, we will describe how to complement the buffers for extracting information about the mitochondrial activity, as well as cell metabolic glucose and HKII dependency, and intracellular K^+^ sensitivity. This analysis is based on the use of 3-bromo-2-oxopropionic acid (3-BP), oligomycin-A, and antimycin-A which either inhibit hexokinase-2 or the ATP-synthase and complex III, respectively. Additionally, gramicidin is used to deplete intracellular K^+^ stores.

7. Complement the buffers with the required compounds.
  a. Pre-warm nutrient-free imaging buffer to 20°C–25°C.
  b. Aliquot 200 mL of buffer in a new bottle without adding additional compounds (= IB -GLU).
  c. Add 10 mM of d-glucose to the residual 800 mL of buffer (= IB + GLU).
  d. Aliquot 200 mL of basal imaging buffer in a new bottle and add 3-BP at a final concentration of 300 μM (= IB + 3-BP) from a 300 mM H_2_O stock (1:1000 dilution).
  e. Aliquot 200 mL of basal imaging buffer in a new bottle and add oligomycin-A and antimycin-A at final concentrations of 3 μM and 5 μM (= IB + O/A) from 10 mM DMSO stocks, respectively (1:3333 and 1:2000 dilutions, respectively).
  f. Aliquot 200 mL of basal imaging buffer in a new bottle and add gramicidin at final concentrations of 15 μM (= IB + GRAM) from a 15 mM DMSO stock (1:1000 dilution).

***Note:*** Other concentrations, modulators, or combinations of metabolic modulators may be used to receive further information about cell metabolic activity.

**CRITICAL:** Ideally, buffers are freshly complemented with glucose and the metabolic modulators (3-BP, oligomycin-A and antimycin-A, and gramicidin) on the day of the imaging experiments due to the potential instability of the compounds in an aqueous solution.

### Preparing the microscope and the perfusion system for the experiments

**Timing: Day 3, 30 min**

Prior to the measurements, the microscope, PC, imaging software, and perfusion system software should be started, followed by the adjustment of the parameters for the subsequent measurements.

8. Start the PC and open the perfusion system software to prepare the perfusion system.
  a. Wash the perfusion system to get rid of any residual components left from older experiments.
    i. Open the number of channels that are necessary for the measurements + one for cleaning, and wash every syringe of the perfusion system with 10 mL of 70% ethanol.
    ii. When the ethanol has passed, add 20 mL of ddH_2_O per syringe to remove residual ethanol.
  b. Close the valves of the perfusion system using the perfusion system software and prepare the system with selected buffers.
    i. Fill one buffer in each of the syringes.
    ii. Shortly open the valves of the perfusion system to remove air from the tubings.
    iii. As the buffers contain toxic components, repeat steps 5.a.i.–5.a.ii. with the channel used for cleaning to remove residual compounds.
    iv. Once all air is removed from the tubes and the system is cleaned, the channel containing the “basal buffer” (IB + GLU) should be opened to prepare the system for the first experiment and the flow rate of the perfusion system should be adjusted to approximately 1 mL/min using tube clamps. Subsequently, perfusion can be stopped by closing all valves in the software.
9. Open the Imaging software and adjust the imaging parameters.
  a. All the sensors presented in this protocol represent FRET-based probes utilizing CFP and YFP, hence, the excitation maximum of all probes is close to 430 nm and emission maxima are close to 475 nm (CFP) and 530 nm (FRET).
    i. Using the described microscope setup, at a binning of 4, a LED intensity at 455 nm of ∼1%–2%, an exposure time of ∼200 ms and an interval time of ∼3 s are good starting points.
    ii. Adjust the settings of the microscope for simultaneous detection of CFP- and FRET emission upon excitation with the 455 nm LED and the 427/10 excitation filter.

***Note:*** We recommend using a 40**×** oil immersion objective to investigate the proper localization of the fluorescent probes upon expression in the mammalian cells.

***Note:*** Light intensities should be minimized to prevent phototoxic effects and photobleaching as much as possible. To maintain suitable fluorescent intensities for measurements, the camera binning can be increased to yield higher fluorescence emission signals from the cells, while keeping excitation light intensities low.

***Note:*** Depending on the expected duration of the experiment, imaging intervals should be adjusted to prevent phototoxic effects and photobleaching as much as possible. For longer measurements (e.g. slow responses/effects), imaging intervals of several seconds (for example 10 s) might be chosen instead of imaging every 3 s.

**CRITICAL:** Although imaging parameters have to be adjusted for every sensor, these parameters should not be changed when measuring the same sensor on a different day. Keeping the same settings for one sensor guarantees consistency (e.g. comparable dynamic range) throughout various measurements (either on the same day or on different days).

### Preparing the experiments

**Timing: Day 3, 10 min**

**Figure 1:**
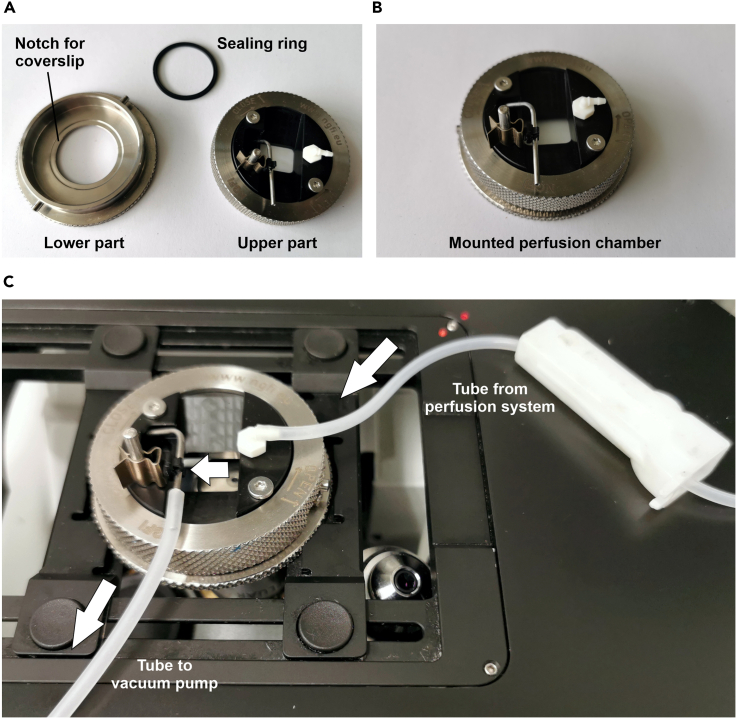
Representation of the perfusion chamber Photograph of the (A) unmounted and (B) mounted perfusion chamber. (C) Displays the perfusion chamber placed on top of a microscope stage. Inlet and outlet of the chamber are indicated.
    iv. Subsequently, close the perfusion chamber by inserting the sealing ring in the upper part of the chamber (Figure 1A) and put the upper part on top of the lower part (Figure 1B). Add 1 mL of the cell equilibration buffer to ensure hydration of the cells.
    v. Tightly close the perfusion chamber by twisting (Figure 1B) and remove excess buffer with a paper towel. Check the tightness of the perfusion chamber by using a fresh, dry paper towel and carefully move around the bottom of the glass coverslip. If the chamber is tight, the towel will remain dry.
  b. Place the perfusion chamber on top of the microscope and start perfusing the cells with IB + GLU.
    i. If using an oil immersion objective, add a drop of fluorescence-suitable immersion oil on the objective.
    ii. Put the perfusion chamber on top of the microscope and fix it (Figure 1C).
    iii. Attach the tube of the perfusion system to the inlet (on one side) of the perfusion chamber (Figure 1C).
    iv. Open the valve of IB + GLU buffer to start perfusing the cells.
    v. Check for functional perfusion (i.e., increasing volume in the chamber) and attach the tube of the vacuum pump to the other side of the chamber (outlet) to prevent buffer overflow and to ensure a laminar flow of the buffer across the coverslip (Figure 1C).
  c. Screen the coverslip for a suitable imaging section with cells showing a good signal to noise ratio and proper targeting of the respective fluorescent probe (Figure 2). Troubleshooting 1, 2 and 3.

10. After 30 min of cell equilibration in the cell equilibration buffer, experiments can be started.
  a. Carefully transfer a glass coverslip containing the cells from the 6-well plate to the perfusion chamber.
    i. A 25G needle and sharp forceps are helpful for the coverslip transfer. Alternatively, a commercially available lens lifter can be used.
    ii. Ensure that no edge of the coverslip is damaged or broken, as this will lead to leakiness of the perfusion chamber.
    iii. Properly place the coverslip in the notch of the lower part of the perfusion chamber (Figure 1A).

**Figure 2:**
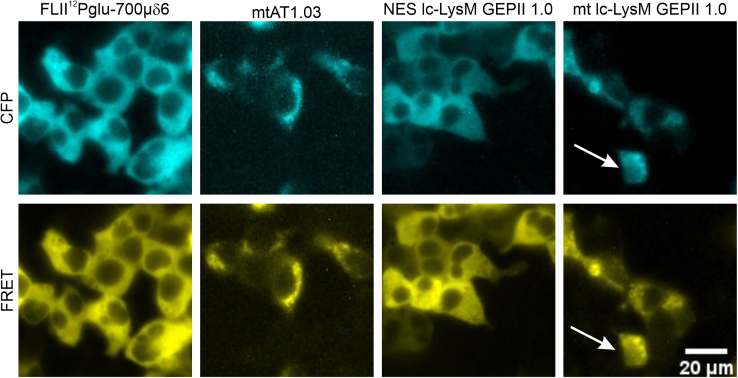
Representative fluorescence wide-field images of HEK293 cells Images display fluorescence wide-field images of the CFP- (cyan, upper images) and FRET-fluorescence (yellow, lower images) of HEK293 cells expressing (from left to right): FLII^12^Pglu-700μδ6, mtAT1.03, NES lc-LysM GEPII 1.0 or mt lc-LysM GEPII 1.0. Arrow indicates a cell where a mitochondrial construct is mistargeted in the cytosol/ nucleus. Scale bar represents 20 μm.
11. Once a section was found, the imaging experiment can be started.

**CRITICAL:** For fluorescent probes targeted to the mitochondrial matrix (mtAT1.03 and mt lc-LysM GEPII 1.0), ensure that cells show a correct targeting of these sensors (solely mitochondrial fluorescence) and do not show cytosolic and/or nuclear mistargeting. We highly recommend performing a preliminary experiment testing for correct targeting and expression of the sensor. Excessive mistargeting can also be caused by the usage of too high DNA or PolyJet transfection reagent concentrations. Also ensure that the cell confluency within the area of interest is not too high, as this will lead to slower cell responses due to limited buffer/compound diffusion.

### Measurement of cytosolic glucose and K^+^ and mitochondrial ATP and K^+^ dynamics

**Timing: Day 3, ∼25 min per experiment**

Upon identification of cells suitable for imaging, the actual experiment can be started.

12. Select your cells of interest and follow the fluorescence over-time of the CFP- and FRET emissions.
  a. Start recording the images at a distinct light intensity, exposure time, binning, and interval. Make sure to not select the brightest cells, as it will then be difficult to keep the settings for the next experiments. Cells possessing average fluorescence intensities that are representative for the whole population might be measured instead.
  b. Draw regions of interest around every cell of interest. Also include one background region, which is important for later analysis. Make sure to select a background region that does not show any fluorescence signal coming from neighboring cells.
  c. Record the baseline of both fluorescence intensities in IB+GLU buffer for 5 min. Troubleshooting 4.
  d. After 5 min, switch to one of the experimental buffers, either containing no glucose (IB -GLU), 3 μM oligomycin-A and 5 μM antimycin-A (IB + O/A), 300 μM 3-BP (IB +3-BP) or 15 μM gramicidin (IB + GRAM).
  e. After switching to the experimental buffers, keep recording the fluorescence over-time values for at least 10 min or until the ratio over-time has stabilized again. Troubleshooting 5.
  f. Perform steps a-e for cells transfected with each sensor (mtAT1.03, FLII^12^Pglu-700μδ6, NES lc-LysM GEPII 1.0, and mt lc-LysM GEPII 1.0, at least 5 times on 3 different days (with the same settings for each sensor) to ensure data consistency and to ensure a stable average for each treatment and sensor (Bischof et al., 2017; Imamura et al., 2009; Takanaga et al., 2008).
  g. After the experiment has finished, stop the image acquisition, export the fluorescence over-time values, e.g., to Microsoft Excel, stop the perfusion system, withdraw the coverslip and extensively wash the perfusion system and -chamber with ethanol and subsequently with ddH_2_O.
  h. Start the next experiment by repeating steps 7–9.

### Analyze data

**Timing: Day 4, 2 h**

After performing the experiments and data export to Microsoft Excel, analysis can be performed for data interpretation and representation.

13. Correct the fluorescence over-time values of every cell measured for the corresponding background intensity.
  a. Subtract the fluorescence over-time of the cyan channel from the CFP signal of the cells and subtract the fluorescence over-time values of the FRET-channel from the FRET signal of the cells.
14. Calculate the FRET-ratio signal by dividing the background-corrected values of FRET/CFP and plot the FRET-ratio signal over-time (Figure 3A).
  a. In case of observing a signal in the FRET-ratio over-time, ensure that the signal is ratiometric, i.e., CFP- and FRET-intensities show opposite responses (Figure 3B). A de- or increase in both channels will most likely point to other effects rather than a FRET change due to binding of the respective ion/metabolite.

**Figure 3:**
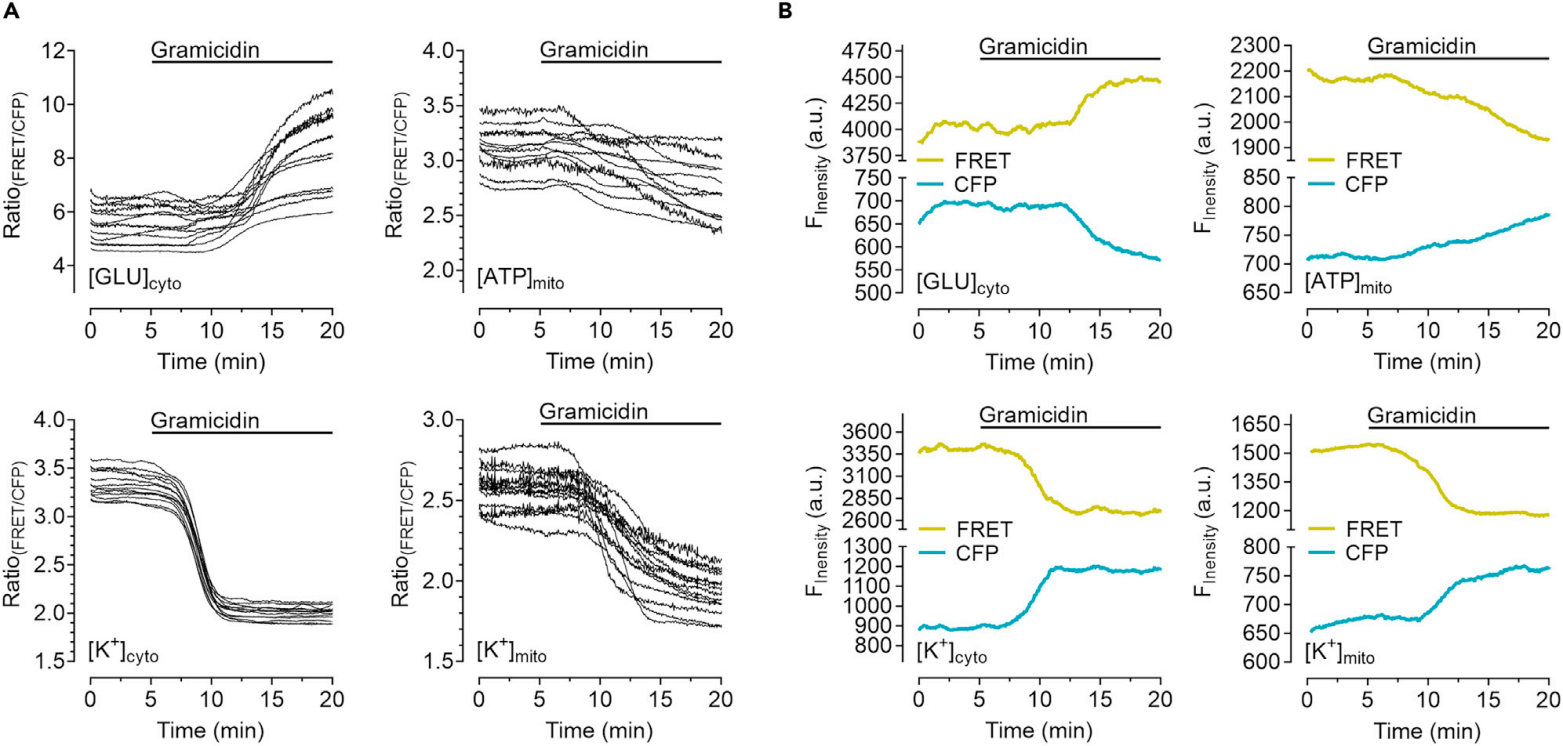
Representative responses of HEK293 cells upon gramicidin treatment (A) FRET-ratio signals over-time of several HEK293 cells either expressing FLII^12^Pglu-700μδ6 (upper left), mtAT1.03 (upper right), NES lc-LysM GEPII 1.0 (lower left) or mt lc-LysM GEPII 1.0 (lower right), respectively, in response to the administration of 15 μM gramicidin at time-point indicated in the panels. (B) Shows the representative single cell fluorescence over-time courses of CFP- (cyan) and FRET-signal (yellow) of the respective FRET-based probes as demonstrated in (A) of one single cell in response to the same treatment.
15. If desired, correct the FRET-ratio over-time for potential fluorescence bleaching effects using the following equation:

$$R_{0}=R_{initial}\cdot \exp\left(-K\cdot Time\right)+R_{plateau}$$

R_0_ = function of the bleaching correction curve

R_initial_ = maximal fluorescence ratio signal once imaging has started

K = rate constant of fluorescence bleaching over-time

R_plateau_ = minimal fluorescence ratio reached by bleaching over-time

**Figure 4:**
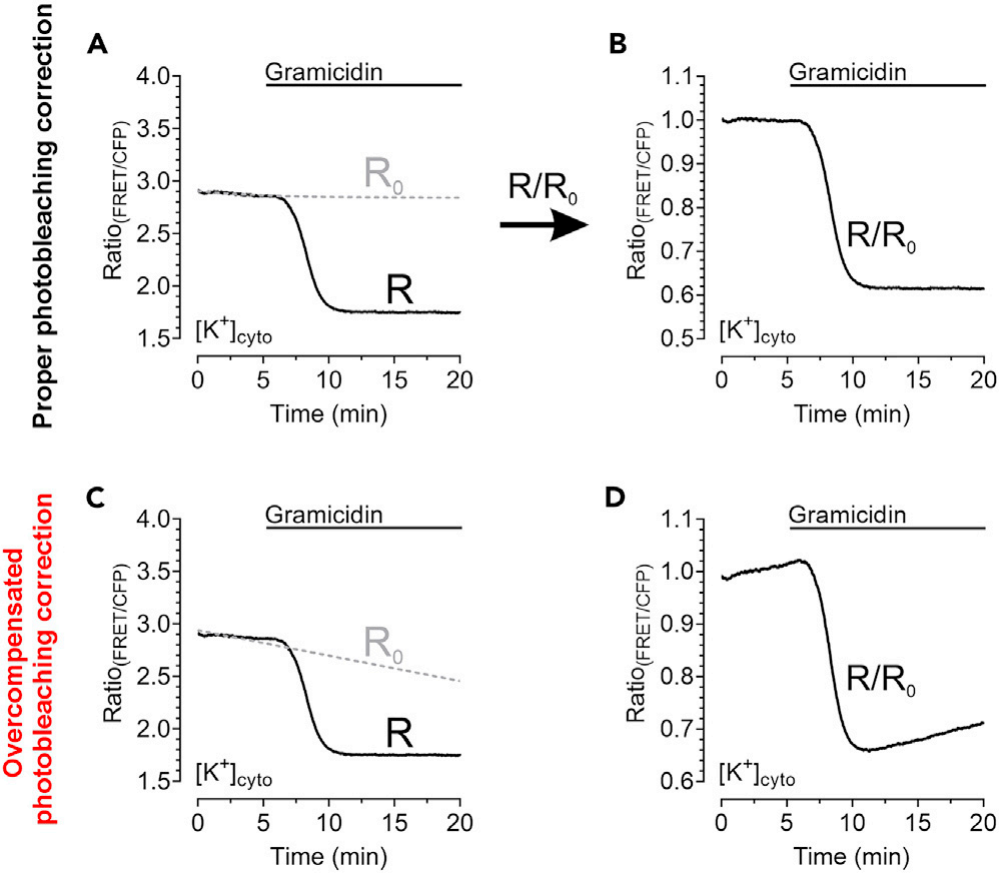
Representative bleaching correction of a single-cell response (A) FRET-ratio signal over-time (R, black solid line) of a HEK293 cell expressing NES lc-LysM GEPII 1.0 in response to gramicidin treatment and the corresponding, proper bleaching correction curve (R_0_, gray dashed line). R_0_ was generated using GraphPad Prism 5 software. (B) Displays the normalized and bleaching corrected FRET-ratio signal over-time of the response demonstrated in (A) as a result of dividing R/R_0_. (C and D) demonstrate an example of an overcompensated bleaching correction of the same curve as demonstrated in (A).

One of the easiest ways to correct for photobleaching using the given formula is to use the one phase decay function provided by GraphPad Prism (Figure 4A).

16. Normalize the data by dividing the FRET-ratio signal by the R_0_ function over-time (Figure 4B) and check the bleaching correction for its correctness. An example of an overcompensated photobleaching correction is demonstrated in Figures 4C and 4D.
17. To receive the metabolic phenotype of cells, plot the average of the normalized [GLU]_cyto_ response of every cell analyzed over the average of the normalized [ATP]_mito_ alteration of every cell analyzed for each of the different treatments. Subtracting the value “1” from both average responses will yield the net change of [GLU]_cyto_ and [ATP]_mito_ from the basal value (Figure 5). Such representation significantly reduces the number of panels and allows to observe the effect of metablic interventions directly at one glance.

**Figure 5:**
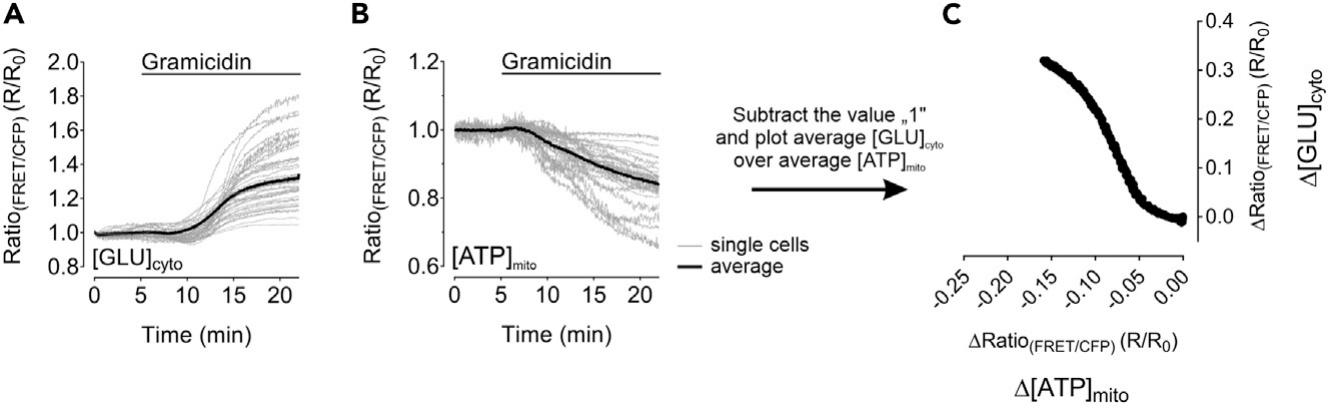
Representative results of [GLU]_cyto_ and [ATP]_mito_ measurements in HEK293 cells upon gramicidin treatment (A and B) (A) [GLU]_cyto_ and (B) [ATP]_mito_ recordings over-time of HEK293 cells in response to treatment with gramicidin at time point indicated in the panels. Single cell responses (gray lines) and average response of the whole cell population (black line) are shown. (C) Displays the result of plotting the averaged [GLU]_cyto_ over [ATP]_mito_ after baseline subtraction. In total, the figure shows the gramicidin sensitivity of [GLU]_cyto_ and [ATP]_mito_ of HEK293 cells in response to gramicidin treatment.
18. To receive the gramicidin sensitivity of the cytosolic and mitochondrial [K^+^] of cells, plot the average of the normalized [K^+^]_cyto_ alteration of every cell analyzed over the average of the normalized [K^+^]_mito_ alteration of every cell analyzed for each of the different treatments by subtracting the value “1” from both average responses will yield the net change of [K^+^]_cyto_ and [K^+^]_mito_ from the basal value (Figure 6). Such representation significantly reduces the number of panels and allows to observe the effect of metablic interventions directly at one glance.

**Figure 6:**
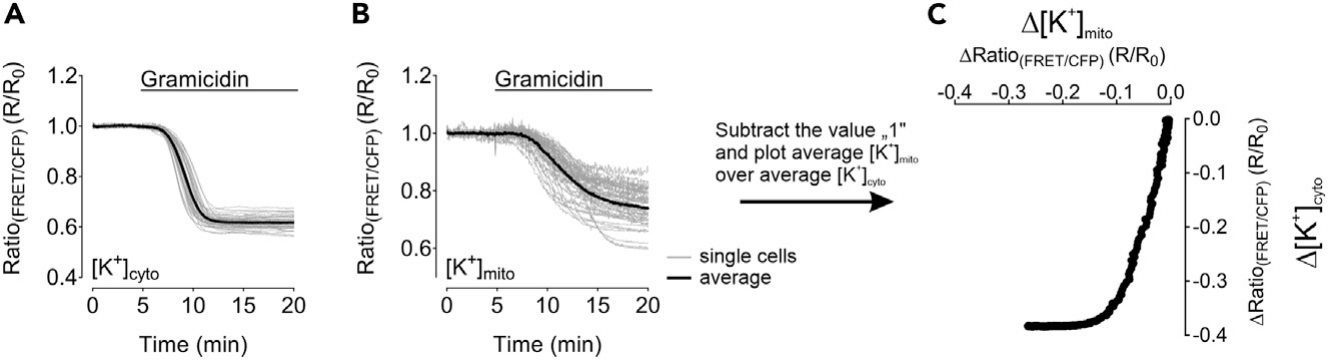
Representative results of [K^+^]_cyto_ and [K^+^]_mito_ measurements in HEK293 cells upon gramicidin treatment (A and B) (A) [K^+^]_cyto_ and (B) [K^+^]_mito_ recordings over-time of HEK293 cells in response to gramicidin treatment as indicated in the panels. Single cell responses (gray lines) and average response of the whole cell population (black line) are shown. (C) Displays the result of plotting the averaged [GLU]_cyto_ over [ATP]_mito_ after baseline subtraction. In total, the shows displays the gramicidin sensitivity of [K^+^]_cyto_ and [K^+^]_mito_ of HEK293 cells in response to gramicidin treatment.

***Note:*** Photobleaching represents an unwanted side-effect. Although it can be reduced to an absolute minimum by adjusting imaging parameters, corrections, as described, may be necessary.

**CRITICAL:** Check the correctness of the photobleaching correction procedure by comparing the uncorrected raw curves/responses with the curves after correction. Bad curve fitting may lead to over- or underestimation of responses/ effects, hence, always double-check. Other corrections than the one-phase-decay might also be tested in case of bad fitting. The exact shape of the plots shown in Figures 5C and 6C requires an extremly thoroughful and exact timing of the experiments. For example, the total duration of the entire experiment as well as the superfusion of the cells with e.g., gramicidin needs to follow identical schedules for all measurements that will be transferred to XY plots.

## Expected outcomes

**Figure 7:**
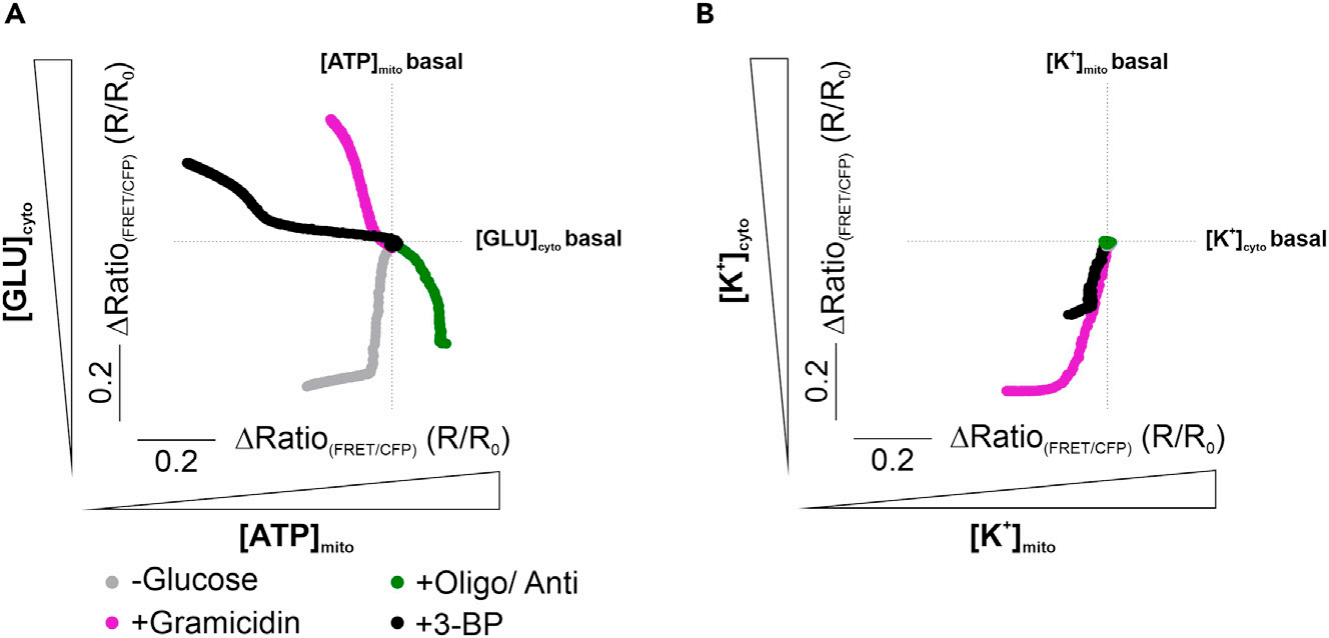
Results of the metabolic- and K^+^ homeostasis phenotyping of HEK293 cells [GLU]_cyto_ and [ATP]_mito_sensitivity (A) and [K^+^]_cyto_ and [K^+^]_mito_sensitivity (B) of HEK293 cells in response to different metabolic interventions as indicated, including the removal of extracellular glucose (-Glucose, gray curves), or the administration of gramicidin (+ Gramicidin, magenta curves), the administration of oligomycin-A and Antimycin-A (+Oligo/ Anti, green curves) or 3-bromo-2-oxopropionic acid (3-BP). Part of the figure has been published in Bischof et al. (2021). Figure reuse with permission from Bischof et al. (2021).

Performing the experiments as described above, will yield the metabolic phenotype of cell lines concerning their glucose and ATP homeostasis and will demonstrate the correlation between glucose/ATP dynamics. Also the other treatments can be represented as described, which will yield the metabolic phenotype of cells (Figure 7A) and the K^+^-sensitivity/phenotype (Figure 7B). Additionally, the experiments will demonstrate mitochondrial and/or cytosolic K^+^ fluctuations in response to the different metabolic interventions and their interrelation.

Besides visualizing metabolite/K^+^ dynamics over-time, already the basal FRET-ratio signal contains information about the basal concentration of the analytes in the cytosol or mitochondria, which can differ significantly between cell types and/ or pretreatment conditions and should therefore also be analyzed separately by comparing the basal values of different treatments/ conditions using appropriate statistical tests.

## Quantification and statistical analysis

Data can be represented and analyzed using any program of choice. We recommend either plotting every single cell response over-time in a separate graph for each treatment or represent data as described above. Experiments should at least be replicated for a minimum of 3 times on 3 independent days to ensure data consistency, reproducibility, and a stable means.

In case of a statistical comparison of treatments is desired, we recommend testing the data for normal distribution to choose the proper statistical test. Usually, an unpaired t-test (if data are normally distributed) or a Mann-Whitney-U test (if data are not normally distributed) should be used for pairwise comparison of two conditions, or a One-Way ANOVA (if data are normally distributed) or Kruskal Wallis test (if data are not normally distributed) should be used for comparison of multiple conditions.

## Limitations

First, due to the use of a perfusion system, cells that should be investigated should show good adherence to the glass coverslip, to not detach them when starting the experiment.

Second, cells that are investigated are exposed to slight shear stress, which can, however, be regulated by adjusting the flow rate. To compare measurements, make sure to adjust the perfusion system to the same speed as a higher speed might lead to faster cell responses and, in turn, to false interpretation.

Third, cells are exposed to potential effects of phototoxicity. Hence, optimizing the imaging parameters in terms of using low light intensities and exposure times may avoid possible toxic side effects caused by excitation light.

## Troubleshooting

### Problem 1

No fluorescence signal is detected when observing the cells under the microscope (steps 10 - 12).

### Potential solution

First, check the imaging settings of the microscope, as something might be wrong with these parameters (e.g., excitation light, filter settings, light path).

Second, check transfection efficiency of your cells of interest using other, in your lab well-known plasmids and eventually check transfection by fluorescence imaging, western blot, qPCR or any other method of choice. If transfection efficiency confirms to be at a low level, try changing to other transfection reagents or use other methods of transduction, for example, viral delivery systems or electroporation.

### Problem 2

Cells detach when perfusion is started (steps 10–12).

### Potential solution

Reduce the flow rate of perfusion. If this does not help and cells still start to detach, try to coat the glass coverslips with a coating reagent of choice. We recommend using poly-L-lysine, for HEK293 cells at a concentration of 0.25 mg/mL.

### Problem 3

Sensors targeted to mitochondria show high levels of mistargeting to the cytosol/nucleus (steps 10 - 12).

### Potential solution

In case of high levels of mistargeting are observed upon expression of mitochondrial targeted biosensors, a change of the transfection reagent or reducing the amount of plasmid used for transfection might be helpful. Reducing the time between transfection and imaging might be also helpful.

### Problem 4

No stable baseline can be recorded, as FRET ratio values are continuously raising or dropping (step 12).

### Potential solution

If no stable baseline appears during imaging, photobleaching may be the reason. Try to optimize the imaging conditions by reducing light intensity, exposure time and/or acquisition intervals.

### Problem 5

Cells do not respond to any treatment (step 12).

### Potential solution

Ensure that cells are not pre-stimulated with any of the compounds used in the perfusion system. Excessive washing of the perfusion system and -chamber may solve the problem.

## Resource availability

### Lead contact

Further information and requests for resources and reagents should be directed to and will be fulfilled by the lead contact Roland Malli, roland.malli@medunigraz.at.

### Materials availability

This study did not generate new unique reagents.

## Data Availability

Original/source data for the paper is available from the lead contact upon request.
